# Secret of Good Looks

**Published:** 1875-10

**Authors:** 


### THE SECRET OF GOOD LOOKS.

In a poem written many years ago by that acute observer of human nature, Prof. Oliver Wendell Holmes, there occurs this couplet:

> "Look to your hat: the secret of good looks
> Lies with the beaver in Canadian brooks."

A thousand times we have heard it said that a man with neat hat and boots and gloves, is well dressed. This can not be very far from the truth. Certain it is that no elegance of costume will suffice to render attractive the wearer of a "shocking bad hat."

For many years we have worn hats made by DUNLAP & Co.; and, to the extent of our head-covering, at least, we have passed for a well-dressed individual. During a long residence in New York, we have learned many things. To other gains we have added a knowledge of the little economies of life. This knowledge has proved useful to us, useful to our friends, useful when we have thought it proper to impart it, to our readers.

An economy which we began to practice long ago, was to buy only the best things. We never had a "cheap" suit of clothes without regretting the fact. We never bought a low-priced hat without afterwards confessing to ourselves that we had erred.

There is no comfort in bad articles of wearing apparel, and there is no satisfaction in them. Especially is this remark true of the hat—the crowning and most conspicuous feature of the dress. It is seen by all, and is the first item of the garb to be examined and criticised. It bears the brunt of storm and dust, and a multitude of hard knocks. If it is faithfully constructed, of good materials, it survives all sorts of usage till the style changes, and perhaps for years afterwards. We know of a Dunlap hat which has been worn in a country town in Massachusetts, by spells, for half a score of years, and still it is not shabby.

No name is better known among hatters than that of Dunlap. The business conducted by what is now the house of Dunlap & Co. was started in 1857 by Mr. Robert Dunlap, who is now the head of a firm embracing several members of the family. It was established on a single idea—that of making the best hat which human skill could fabricate. This original thought has been adhered to amid all vicissitudes. The war and its great attendant rise in the cost of materials and labor, was not able to lower the standard of the Dunlap hat. It maintained its superiority amid the reign of shoddy and the cheapening processes to which manufactured goods were subjected. Then, as now, styles were originated and fashions determined upon by the Dunlaps. This power they have ever used without abusing it; for, in four days after they announce a new style, they allow the block upon which it was made to be sold to the trade.

They make many hats, but they give their name to but one grade—the best. What is called the "silk hat" is not infrequently made upon a basis of muslin, rendered stiff by gum shellac. The first injury inflicted upon such a hat destroys its symmetry forever. A genuine "Dunlap" is certain to have a "fur body." This makes a hat which will wear. You may dent it and break it, and sit on it, but you can not ruin it.

The great factory of the Dunlaps, on Mercer Street, turns out an enormous number of hats each year. The styles comprise the fashionable "stove-pipe," in various colors; felt hats, straw hats, and opera-hats. These last are a jointed framework, over which merino, felt, and satin, is shaped. When on the head it is a neat high hat. In a second of time it can be flattened to the thickness of a dinner-plate. These are much worn by frequenters of parties and places of amusement. Agents sell the Dunlap hats in all the chief cities of America, and the price and quality is everywhere the same. This is an inflexible rule, and one which is never deviated from.

There is a class of mechanics who hive in cellars in the large cities, and offer hats at low prices. They are known among hatters as "skinners," or "resurrectionists." Hatters allow a trifle for old hats, which they sell to this class. The underground artist strips off the worn covering, blocks over the body, inverts the silky envelope; concealing under the band the portion which formerly extended around the crown; glues a little silk plush upon the badly worn spots, trims it up, and offers you your old hat, remodelled, for $4 or $5. Of course you do not buy a very choice hat when you purchase such a re-animated body. But it is curious to know that there is a vast difference in the value of these worn-out frames which the humble artizan thus raises from the dead. Ask any one of these men what worn-out hat gives him the best material for re-habilitation, and he will reply, "a Dunlap, by all odds." This is a compliment to the materials used and the care exercised by this house.

The Messrs. Dunlap do not advertise in the public prints, and may not thank us for even this much of kindly publicity. That "their works do praise them," is evident from the enormous patronage which they enjoy. Their stores at 174 Fifth Avenue, near Twenty-Third Street, and 589 Broadway, are usually thronged with customers; and the class of trade which they enjoy is, without doubt, the highest in the country.

